# NO_2_ exposure increases eczema outpatient visits in Guangzhou, China: an indication for hospital management

**DOI:** 10.1186/s12889-021-10549-7

**Published:** 2021-03-15

**Authors:** Luwen Zhang, Dian Jing, Qiaochu Lu, Shuqun Shen

**Affiliations:** 1 School of Health Services Management, Southern Medical University, Guangzhou, 510515 Guangdong China; 2 Department of Biostatistics, School of Public Health, Southern Medical University, Guangzhou, China; 3 Dermatology Hospital, Southern Medical University, Guangzhou, 510000 China

**Keywords:** Eczema, NO_2_, Outpatient visits, Management

## Abstract

**Background:**

Ambient nitrogen dioxide (NO_2_) is a common air pollutant in developing countries and causes skin conditions, but its effect on eczema in subtropical areas is not clear in China.

**Object:**

To measure the effect of short-term exposure of NO_2_ on the incidence of eczema and the change of outpatient visits.

**Methods:**

Data of daily temperature, air pollutants, and outpatient visits from 2013 to 2018 were collected in a row. The generalized additive model (GAM) and Poisson distribution were used to assess the association between short-term exposure of NO_2_ and the outpatient visits of patients with eczema. The cumulative exposure effect of lag 0–3 days and the displacement effect of NO_2_ and other pollutants were considered as well. A single pollutant model was used to examine the independent association, and a two-pollutant model was adopted to control the confounding effect.

**Results:**

The daily outpatient visits of eczema increased from 75.26 to 190.85 from 2013 to 2018 (*P* < 0.001). The combined influence of NO_2_ and the related pollutant exerted a stronger influence on the incidence of eczema. The maximum effect of NO_2_ appeared on the exposed day.

(lag 0) and disappeared on day 4 (lag 3). The children and seniors were more vulnerable to NO_2_ exposure.

**Conclusion:**

Exposure to NO_2_ is tightly associated with eczema incidence and outpatient visits. The hospitals should react to the visit fluctuations and adjust physician duty shifts to improve outpatient service efficiency.

**Supplementary Information:**

The online version contains supplementary material available at 10.1186/s12889-021-10549-7.

## Introduction

Eczema, or atopic dermatitis, is a chronic, relapsing, and severely pruritic skin disorder [1]. It is a common condition at any age, affecting approximately 10–20% of children, and 2–18% of adults worldwide [2]. The prevalence of eczema has been increasing, mostly prevalent in industrialized countries, and closely followed by the developing countries [3]. Eczema has a significant impact on the quality of life and has been increasingly considered a public health problem [4]. The possible causes of eczema include genes, dry skin, immune system or mental problems, and the most important—triggers in the environment, such as low humidity, heat, sweating, as well as air pollutants [5].

The recent studies have just expanded to the influence of nitrogen dioxide (NO_2_) on eczema incidence. NO_2_ is a toxic reddish-brown gas, a strong oxidizing agent and common atmospheric pollutant that is produced by combustion, as of fossil fuels, car exhaust, smog, and cigarettes. Since the concentration of NO_2_ is tightly related to industry development, manufacturing, and transportation, it is a common air pollutant in developing countries and regions that pursue industrialization [6]. The inhale of NO_2_ damages the respiratory system, and the skin exposure also damages skin integrity and makes eczema happen [7]. The association of ambient air pollution, meteorological factors, and outpatient visits for eczema has been studied in some limited regions in China, such as Shanghai [8]. Currently, there has not any research done in the southeastern subtropical Guangdong Province. The climate is very different between Guangdong in the subtropical zone and Shanghai the temperate zones. In addition, the pollution control measures implemented by the Chinese government has significantly changed the air pollution distribution since 2015. It requires update data to illustrate the new changes.

Eczema, as a common but relapsing condition, has a comparatively stable and repeated “diagnosis—treatment—follow-up—education—readmission” treatment process [9]. If the ambient NO_2_ is associated with the increased eczema incidence, there may accompany the burst of outpatient visits. It may cause over-crowding at hospital outpatient and emergency departments (OEDs), and require on the outpatient service process arrangement and physician’s on-duty shifts [10]. If the hospital can react to the change of weather and air pollutants, it will improve both the efficiency and patient satisfaction. The study is to discuss how the findings could be used in the management of eczema patients in the outpatient department as well.

## Methods and materials

### Study setting

Guangdong Province is located in Southeastern China, with latitude 23.02 N, and longitude113.75E. It has a humid subtropical to tropical climate with an annual average temperature of 22 °C (72 °F) and sharp seasonal change of humidity from over 95% in summer to lower than 40% in winter. Guangzhou city, the capital of Guangdong province, locates in the geometrical center of the province, has the typical climate characteristics. Meanwhile, as the local industry and trade center, it suffers the heaviest air pollution. There lacks evidence of how the weather and air pollution affect the prevalence of eczema in Guangdong province.

The study site, Guangdong Dermatology Hospital, is the only provincial-level research center on dermatology in Guangdong province as well as tertiary-level teaching hospital affiliated to Southern Medical University. The hospital is located in Guangzhou City, but it provides outpatient care to patients from the whole province.

### Data

We obtained data of daily outpatient visits from Guangdong Dermatology Hospital. Patients who were diagnosed with eczema (IDC-10 code L30.902) were included from January 18, 2013, to December 31, 2018. If the same patient re-visit the outpatient department after the first diagnosed eczema within 21 days, we defined the series of visits as one single visit in accord with the insurance policy.

We included 5 pollutants adopted in this research: NO_2_, sulfur dioxide (SO_2_), particulate matter no greater than 10 μm (PM_10)_, particulate matter no greater than 2.5 μm (PM_2.5_), and daily maximum 8-h ozone (O_3_). The daily average concentrations of air pollutants were obtained from the public sharing system of Guangzhou Environmental Monitoring Center (http://112.94.64.160:8023/gzaqi_new/RealTimeDate.html). The system synthesized data from 11 national air quality monitoring stations in Guangzhou city, and took the average of all monitoring values. The daily relative humidity and temperature data were obtained from the China Meteorological Data Sharing Service System. The air pollution and meteorology data followed the quality control programs which were mandated by the Chinese government.

The air pollution and meteorological data were ranged from January 18, 2013, to December 31, 2018. Data sources are public and widely used in related studies [11].

### Statistical methods

The generalized additive model (GAM) and Poisson distribution were used to assess the association between short-term exposure of NO_2_ and the outpatient visit of patients with eczema. The cumulative exposure effect of lag 0–3 days, and the displacement effect of NO_2_ and other pollutants were considered as well.

The single pollutant model was used to examine the independent association, and the two-pollutant model was adopted to control the confounding effect of the other pollutants. The model was expressed as follows:

$$ {\displaystyle \begin{array}{l}\log \left({\mu}_t\right)=\alpha +\sum {\beta}_i{X}_{ti}+ NS\left({temperature}_t, df=3\right)+ NS\left({humidity}_t, df=3\right)\\ {}+ NS\left({ ti me}_t, df=10/ year\right)+\gamma {DOW}_t\end{array}} $$

where *m*_*t*_ is the expected eczema outpatients on day *t*; is the intercept; *X*_*t*_ is the concentrations of pollutant (NO_2_, SO_2_, PM_2.5_, PM_10_, and O_3_) with *i* = 1 in single pollutant model and 2 in two pollutants model respectively; *b*_*i*_ stands for the coefficient for *X*_*i*_. A natural cubic spline function (NS) with three degrees of freedom (df) were applied for temperature and humidity to capture the nonlinear relationships of time trend [12]. An NS with 10 df per year is used for calendar time to adjust for long-term trend and seasonality [13]. Day of week (*DOW*_*t*_) was set in the form of categorical variables in the model, and *γ* is the effect of *DOW*_*t*_ on eczema outpatients [14].

Besides, we conducted stratification analyses by potential individual-level effect modifiers, including gender and age (< 12 years, ≥12 years and < 65 years, ≥65 years) using the above basic models. To find the best degree of freedom used in the above model, the Akaike information criterion for quasi-Poisson (Q-AIC) was employed in the assessment of the goodness of model fits among 3–10 (per year) df for temperature, humidity, and time respectively [13, 15]. The minimum value of Q-AIC represented the best goodness and the optimum df. We also conducted sensitivity analyses to check the robustness of our modeling strategies by changing the df for temperature (2–4), humidity (2–4), and calendar time (9–11 per year) to control the time trend. We excluded holiday effect since there is no holiday breaks in the study hospitals and the number of patient visits was similar between weekdays and holidays.

R software version 4.0.2 was used to conduct all the analyses. *P*-value < 0.05 was considered to be statistically significant for all statistical tests.

## Results

**Fig. 1:**
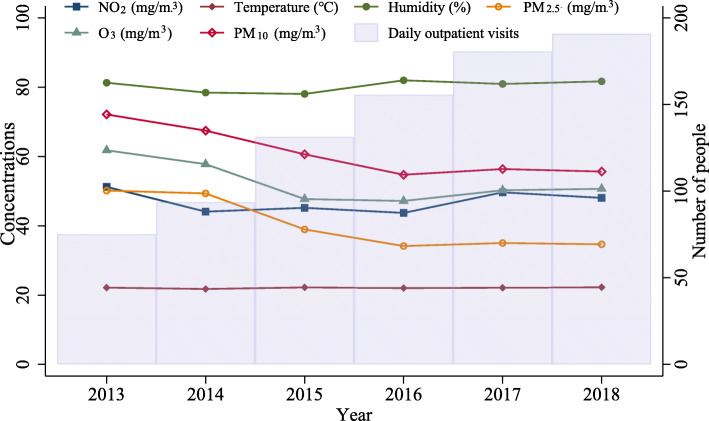
The distribution of air pollutants, temperature, and relative humidity in Guangzhou city, 2013–2018

The average concentration of pollutants varied during the time: the concentration of NO_2_, O_3_, and PM_10_ decreased from 2013 to 2016, but increased from 2017 to 2018; while PM_2.5_ and SO_2_ kept decreasing from 2013 to 2018, indicating a significant improvement in air quality. The humidity ranged from 78.057 ± 8.907% in 2015 to 81.991 ± 9.698 in 2016 (*P* < 0.001), but the average ambient temperature was stable around 22 °C (*P* = 0.905), and the comparative humidity fluctuated around 80% (*p* < 0.001) in the 6 years. (Fig. 1) The daily outpatient visits for eczema increased significantly from 75.26 in 2013 to 190.85 in 2018 (*P* < 0.001). There were more male patients than female, and the trend is kept over time (P < 0.001). The majority of patients were aged 12–65 from 2013 to 2018 (P < 0.001). (Table 1).

**Table 1:** The daily outpatient visits for eczema, age and gender distribution: in Guangdong Dermatology Hospital, 2013–2018

	2013	2014	2015	2016	2017	2018	***F/*** *χ*^2^	***P***
**Daily outpatient visits**	75.261 ± 23.840	93.688 ± 31.839	131.406 ± 37.233	155.644 ± 43.374	180.638 ± 48.581	190.845 ± 48.430	478.389	<.001
**Sex**								
***Male***	13,315 (9.0%)	17,361 (11.8%)	22,967 (15.6%)	27,441 (18.6%)	32,839 (22.2%)	33,709 (22.8%)	49.927	<.001
***Female***	12,349 (8.5%)	16,553 (11.4%)	23,025 (15.8%)	27,190 (18.7%)	32,551 (22.3%)	34,040 (23.4%)		
**Age**	41.530 ± 21.170	42.166 ± 21.059	42.948 ± 20.796	42.134 ± 20.732	41.772 ± 21.107	41.684 ± 20.946	26.794	<.001
**< 12**	2179 (9.0%)	2769 (11.4%)	3509 (14.5%)	4388 (18.1%)	5754 (23.8%)	5587 (23.1%)	89.416	<.001
**12–65**	18,039 (8.8%)	23,806 (11.6%)	32,127 (15.6%)	38,347 (18.6%)	45,546 (22.1%)	47,942 (23.3%)		
**> 65**	5446 (8.6%)	7340 (11.6%)	10,356 (16.3%)	11,896 (18.8%)	14,091 (22.2%)	14,221 (22.4%)		

The concentration of NO_2_ was negatively related to the temperature but positively related to the concentration of PM_2.5_, O_3_, PM_10,_ and SO_2_ (Table 2). The highest correlation was found between NO_2_ and PM_2.5_ and PM_10_ (Pearson correlation 0.73, 0.8, *P* < 0.001).

**Table 2:** The linear correlation of NO2 and temperature, relative humidity and air pollutants by Pearson analysis

	Temperature	Humidity	PM_2.5_	O_3_	PM_10_	SO_2_	NO_2_	CO
Temperature	–	0.31^**^	−0.32^**^	0.20^**^	−0.24^**^	− 0.15^**^	− 0.27^**^	− 0.15^**^
Humidity	–	–	−0.22^**^	−0.41^**^	− 0.26^**^	−0.17^**^	0.02	−0.00
PM_2.5_	–	–	–	0.31^**^	0.94^**^	0.60^**^	0.73^**^	0.18^**^
O_3_	–	–	–	–	0.35^**^	0.26^**^	0.11^**^	0.00
PM_10_	–	–	–	–	–	0.44^**^	0.80^**^	−0.05^*^
SO_2_	–	–	–	–	–	–	0.27^**^	0.68^**^
NO_2_	–	–	–	–	–	–	–	−0.14^**^

^*^p-value < 0.05, ^**^p-value < 0.0001

The smallest Q-AIC was observed in 3, 3, 10 per year df for temperature, relative humidity, and time respectively. Table 3 reported the lag nonlinear model analysis for NO_2_. The maximum effect of NO_2_ was at lag 0 day. Every 10 μg/m3 increase of NO_2_ was associated with a 4.10 percentage (95%CI, 3.80–4.40%) increase of eczema outpatients. The risk decreased gradually from 3.31% (95%CI, 2.99–3.64%) in lag 1 day to 1.64% (95%CI, 1.33–1.95%) in lag 2 day, and then faded away in lag 3 day.

**Table 3:** The RR and PC of eczema risk associated with 10 mg/m^3^ increase of NO^2^

Pollutant (per 10 μg/m^**3**^)	Lag Day	RR	PC (%)	***P***-value
NO_2_	Lag 0	1.0410 (1.0380–1.0440)	4.10 (3.80–4.40)	<.001
	Lag 1	1.0331 (1.0299–1.0364)	3.31 (2.99–3.64)	<.001
	Lag 2	1.0164 (1.0133–1.0195)	1.64 (1.33–1.95)	<.001
	Lag 3	1.0057 (1.0027–1.0087)	0.57 (0.27–0.87)	0.056

Note: *PC* refers to percentage change

The combined influence of NO_2_ and the other related pollutant exert a stronger influence on the incidence of eczema. The combination with SO_2_, O_3_, PM_10_ and PM_2.5_ lead to 4.26% (3.9–4.62), 2.04%(1.85–2.23), 1.75% (1.58–1.92), and 2.01% (1.8–2.23) increase of eczema outpatient visits (Table 4). (*P* < 0.001 for all).

**Table 4:** The RR and PC of eczema risk associated with 10 μg/m^3^ increase of NO^2^ under a two-pollutant model

Pollutant (per 10 μg/m^**3**^)	Model type	RR	PC (%)	***P***-value
NO_2_	NO_2_ + SO_2_	1.0426 (1.039–1.0462)	4.26 (3.9–4.62)	<.001
	NO_2_ + O_3_	1.0204 (1.0185–1.0223)	2.04 (1.85–2.23)	<.001
	NO_2_ + PM_10_	1.0175 (1.0158–1.0192)	1.75 (1.58–1.92)	<.001
	NO_2_ + PM_2.5_	1.0201 (1.018–1.0223)	2.01 (1.8–2.23)	<.001

Note: *PC* refers to the percentage change

The short-term exposure of NO_2_ exerted different influences on patients of different gender and age. The exposure was associated with increased outpatient visits for both male and female for 3 days from lag 0 day to lag 2 day, with PC 4.14(3.83–4.46) to 1.59(1.25–1.92) for male and 4.03(3.7–4.36) to 1.67(1.33–2.02 for female (*P* < 0.001). The children aged< 12 reported the highest risk in the lag 0 day and lag 1 day PC [7.22% (6.62–7.81), 5.05% (4.41–5.7%), P < 0.001], followed by seniors ≥65 years [4.93(4.46–5.41), 3.69(3.16–4.21), *P* < 0.001]. (Table 5).

**Table 5:** Stratification analysis of the percent change of eczema risk associated with 10 mg/m^3^ increase of NO^2^

		Male		Female		< 12		12–65		≥65	
**Pollutant** **(per 10 μg/m**^**3**^**)**	**Lag Day**	**PC (%)**	***P*-value**	**PC (%)**	***P*-valu**	**PC (%)**	***P*-valu**	**PC (%)**	***P*-valu**	**PC (%)**	***P*-valu**
NO_2_	Lag 0	4.14 (3.83–4.46)	<.001	4.03 (3.7–4.36)	<.001	7.22 (6.62–7.81)	<.001	3.54 (3.24–3.84)	<.001	4.93 (4.46–5.41)	<.001
	Lag 1	3.35 (3.01–3.7)	<.001	3.23 (2.87–3.59)	<.001	5.05 (4.41–5.7)	<.001	2.96 (2.64–3.28)	<.001	3.69 (3.16–4.21)	<.001
	Lag 2	1.59 (1.25–1.92)	<.001	1.67 (1.33–2.02)	<.001	1.1 (0.49–1.71)	0.07	1.62 (1.31–1.93)	<.001	1.64 (1.14–2.14)	0.001
	Lag 3	0.4 (0.09–0.72)	0.201	0.73 (0.4–1.05)	0.027	−1.13(− 1.7 - -0.56)	0.05	0.64 (0.35–0.94)	0.029	0.86 (0.39–1.35)	0.071

Note: *PC* refers to the percentage change

The effect of combinations of pollutants were reported in Table 6. The combination of NO_2_ and SO_2_ reported the highest effect than other combination for all age and gender groups, and was followed by the combination with PM_2.5,_ PM_10_ and O_3._ The female group reported slightly higher PC than male group, and the children aged< 12 and the senior aged≥65 reported higher PC than those aged 12–65.

**Table 6:** Stratification analysis of the percent change of exposure risk associated with 10 mg/m^3^ increase of NO^2^ in two-pollutant combinations

Pollutant (per 10 μg/m^**3**^)	Model type	Male	Female	< 12	12–65	≥65
NO_2_	NO_2_ + SO_2_	4.21 (3.83–4.59)	4.27 (3.88–4.67)	5.55 (4.84–6.27)	3.87 (3.51–4.23)	5.02 (4.44–5.6)
	NO_2_ + O_3_	2.04 (1.8–2.29)	2.18 (1.93–2.44)	3.76 (3.31–4.22)	1.83 (1.6–2.05)	2.67 (2.3–3.05)
	NO_2_ + PM_10_	2.25 (2.0–2.51)	2.42 (2.15–2.68)	3.38 (2.9–3.87)	2.07 (1.84–2.31)	3.03 (2.64–3.41)
	NO_2_ + PM_2.5_	2.46 (2.12–2.79)	2.6 (2.25–2.94)	3.71 (3.09–4.34)	2.22 (1.91–2.54)	3.65 (3.15–4.16)

Note: *PC* refers to the percentage change

## Discussion

Our study has found that short-term exposure to ambient NO_2_ is positively associated with the significant increase of the outpatient visits for patients with eczema. The detrimental effect lasted for 2–3 days after exposure, and is reinforced when combined with other air pollutants like SO_2_ and PM_2.5_. The exposure is risk factor for both genders and for.

The findings in this study are consistent with the previous findings. The NO_2_ has been a proven risk factor for eczema in countries at all development levels, including the US [16], China [8], Japan [17], and Belarus [18]. Most research on NO_2_ risks have been done in Shanghai. Li et al. reported an association of 10 μg/m3 increase of 7-day (lag 06) average concentrations of NO_2_ was associated with2.22% (95% CI: 1.27, 3.16%) and 2.31% (95% CI: 1.17, 3.45%) increase in outpatient visits for eczema in Shanghai between 2007 to 2011 [19]. Liu Wei also found a strong association between childhood atopic eczema and the increments of NO_2_ in the approximate interquartile range (20 μg/m3) during the gestational period in Shanghai from 2011 to 2012 [20]. Our findings showed that Guangzhou city, as the economic and industrial center of South China, is facing increasing demand of eczema treatment from 2013 to 2018, indicating that eczema has become a public health concern and needs to treated seriously. The government should pay attention to air pollutants like NO_2_ and take measures to manage and control the emissions.

Our study also proved that the combined effect of NO_2_ and other air pollutant factors exerted attenuated effect on eczema, indicating that the mixture may represent a higher risk than the individual component. The consistent associations have also been proven by previous studies. In western China’s Chengdu city, the combined effect of NO_2,_ SO_2_ and PM_10_ showed increased percentage changes in daily outpatient visits for eczema than single effect of NO_2_ [21]. The study in Beijing reported consistently significant positive associations in two pollutant models, but the effects were lower than those in the single pollutant models [22]. The attenuated risks may be explained by the mechanism of how ambient air pollutants cause adverse effects on skin health. There are 4 generally acknowledged mechanism (a) oxidative stress, (b) alterations of microflora, (c) activation of the aryl hydrocarbon receptor (AhR), and (d) induction of the inflammatory cascade and subsequent impairment of the skin barrier [23]. The mixture of pollutants may activate several pathways simultaneously. For example, SO_2_ may enhance the production of reactive oxygen species, and further reduces the content of antioxidants in the skin [24]. PM_10_ may settle on the skin, blocking pores and therefore creating an anaerobic environment for bacteria strain responsible for acne [25]. It would be easier for the negative effect work together to damage the skin function and cause eczema.

We also found children and seniors are the vulnerable population. There are many findings on the eczema incidence for infants and children worldwide [26–28]. The infants and children are eligible to eczema due to the special skin structure and immature immune system, and the comprehensive and systemic measures should be taken to offer careful protection and cares to children [29]. The seniors are vulnerable due to the fragile skin and decreased immune system [30]. The gender-difference is not very significant in our finding, and both genders reported similar effective powers. As for the protective measures for the vulnerable population, the integration of health education and medical care would be more effective. Among all intervention plans, the multidisciplinary age-related structured training educational programs [31], nurse-led workshops, interventions, and care programs [32], as well as structured self-management education training programs [33] are proved efficient in reduction of severity of the condition, improving patient health outcome, and improving coping behaviors.

The lagged accumulative effects showed a sharp rise of outpatient visits in the following 3 days after the short-term NO_2_ exposure. The highest number of visits come at the current day (lag 0), and decreased gradually in the following 2 days (lag 1 and 2), and turns back to normal on the third day. This change will lead to service burden for physicians serving outpatient and emergency department. This finding is very indicative of the management of the outpatient department of the hospital. AHRQ has developed a series of guides for hospitals on improving the management of outpatient visit flows and reducing emergency department crowding [6]. The hospital of dermatology, as the specialized hospital, should develop an alert, flexible, and fast-reacting system which can predict/estimate the weather and pollution change to maintain high efficiency for the treatment of the common and reemerging conditions [34]. There are a bunch of plans to adopt, such as open a green channel for eczema, set up a standard service package, provide bundled service process, et al. The schedule of human resources is the most important task [35]. Since eczema is mild but relapsing condition that periodically influenced by air pollutants, it might be a proper measure to reorganize an integrated care process and manage the pre-and after outpatient management to serve the patients’ demand for services in hospitals. The integration of information technology and service process can improve the efficiency of patient management, measures include establishing patient files, sending weather alert to patients through social or hospital apps, make follow-up or revisit online, or scheduling the appointment on WeChat, et al.

Despite the organization management strategies, hospitals should cooperate with weather report departments, getting informed and alerted to certain pollutants, and should adopt predictive models to estimate the peaks and valleys of patient visits. Hospitals should manage patients waves, adjust the number of physicians on outpatient duty accordingly, and even consider the opening of eczema outpatient clinic in some certain season. Although we did not make predictions in this study, there are researchers devoted to predictive studies. Gul and Celik made an exhaustive review and analysis on the statistical forecasting methods in the forecasting of hospital emergency departments (EDs) [36]. Yucesan et al. developed a multi-method patient arrival forecasting outline for EDs, combined ARIMA, ANN, and the hybrid methods of the two [37]. The predictive methods can offer reliable evidence for decision-making in hospitals, and will be used in our further research.

## Conclusion

The prevalence of eczema has been increasing since 2013 in Guangzhou city and has become a public concern now. The incidence of eczema is tightly associated with the concentration of NO_2_ and causes the changed patients to flow to outpatient visits. The hospital managers should attach great importance to disease management, hospital resource arrangement with climate change, and take measures to develop an integrated and multidisciplinary process to improve disease and hospital management.

### Limitation

First, this study focused on the explored the influence of NO_2_ on eczema, and merely made shallow discussion about the interaction effects between NO_2_ and some major air pollutants. We did not include the influence of temperature and humidity, which might be also important to the incidence of eczema. Second, we used a comparatively simple mathematical model to evaluate the increase of outpatient visits in the future instead of using more advanced prospective analysis methods such as machine learning. Instead, we have the plan to made the unfinished analysis in the following studies. Third, the predictive analysis of hospital management needs more concrete data on physicians, patients, and resources, which are not sufficient in this research but will be our research direction for the further stage.

## Supplementary Information

**Additional file 1.** The distribution of major variables: daily hospital admissions, weather, air pollution and particles in Guangzhou from March 1, 2013 to Dec. 31, 2018 (mean ± SD, anova test).

## Data Availability

The air pollution data that support the findings of this study are available on request from the corresponding author, Shuqun Shen. The atopic dermatitis outpatient visits data are not publicly available due to the privacy restriction.
