# Supplementary material for: NO2 exposure increases eczema outpatient visits in Guangzhou, China: an indication for hospital management
Source: BMC Public Health. 2021 Mar 15;21:506. doi: 10.1186/s12889-021-10549-7 (PMC7962398; doi:10.1186/s12889-021-10549-7)
Supplement: Supplementary file 1 — Additional file 1. The distribution of major variables: daily hospital admissions, weather, air pollution and particles in Guangzhou from March 1, 2013 to Dec. 31, 2018 (mean ± SD, anova test). [file 12889_2021_10549_MOESM1_ESM.docx]

The distribution of major variables: daily hospital admissions, weather, air pollution and particles in Guangzhou from March 1, 2013 to Dec. 31, 2018 (mean±SD, anova test)

|  | **2013** | **2014** | **2015** | **2016** | **2017** | **2018** | | ***F*** | ***P*** |
| --- | --- | --- | --- | --- | --- | --- | --- | --- | --- |
| Daily outpatient visits | 75.261±23.840 | 93.688±31.839 | 131.406±37.233 | 155.644±43.374 | 180.638±48.581 | 190.845±48.430 | 478.389 | | <.001 |
| NO_2_ (μg/m^3^) | 51.237±21.476 | 44.093±18.826 | 45.188±17.063 | 43.716±18.109 | 49.664±19.595 | 48.064±19.605 | 9.315 | | <.001 |
| Temperature (℃) | 22.160±5.585 | 21.768±6.580 | 22.229±5.927 | 22.019±6.395 | 22.136±5.837 | 22.255±6.177 | 0.313 | | 0.905 |
| Humidity (%) | 81.296±11.546 | 78.448±10.628 | 78.057±8.907 | 81.991±9.698 | 80.942±11.373 | 81.685±10.203 | 9.472 | | <.001 |
| PM_2.5_ (μg/m^3^) | 50.165±27.241 | 49.350±24.731 | 38.958±21.112 | 34.163±17.220 | 35.033±19.055 | 34.663±21.135 | 40.575 | | <.001 |
| O_3_ (μg/m^3^) | 61.797±36.736 | 57.796±33.119 | 47.715±24.465 | 47.175±24.994 | 50.253±27.124 | 50.678±25.623 | 14.398 | | <.001 |
| PM_10_ (μg/m^3^) | 72.139±34.403 | 67.476±30.655 | 60.636±28.122 | 54.739±25.081 | 56.388±27.007 | 55.658±27.538 | | 21.298 | <.001 |
| SO_2_ (μg/m^3^) | 20.798±8.447 | 20.953±15.474 | 12.998±5.216 | 11.954±3.783 | 11.739±3.645 | 9.603±3.262 | | 135.621 | <.001 |
